# Comparison between round multi-strand wire and rectangular wire bonded retainers: a randomized clinical trial

**DOI:** 10.1590/2177-6709.28.2.e2321101.oar

**Published:** 2023-05-19

**Authors:** Emad F. AL-MAAITAH, Sawsan ALOMARI, Kazem AL-NIMRI

**Affiliations:** 1 Jordan University of Science and Technology, Faculty of Dentistry, Department of Preventive Dentistry, Orthodontics (Irbid/Jordan).

**Keywords:** Bonded retainers, Gingival health, Relapse

## Abstract

**Objective:**

The primary objective was to compare round multi-strand wire and Ortho-Flex-Tech™ rectangular wire retainers in terms of gingival health. The secondary objectives were to assess plaque/calculus accumulation, and to determine the effectiveness of these retainers in maintaining tooth alignment and their failure rate.

**Material and Methods:**

This single-center study was a two-arm parallel randomized clinical trial and was conducted at the Orthodontic clinics in Dental Teaching Center/Jordan University of Science and Technology. Sixty patients, with bonded retention for the mandibular anterior segment after fixed orthodontic treatment, were randomly selected. The sample comprised Caucasian patients with mild to moderate pretreatment crowding in the mandibular anterior region, Class I relationship, treated without extraction of mandibular anterior tooth. In addition, only patients presenting normal overjet and overbite after treatment were included.

**Intervention:**

One group received round multi-strand wire retainer (30 patients, average age: 19.7 ± 3.8 years), while the other group received Ortho-Flex-Tech™ retainer (30 patients; average age: 19.3 ± 3.2 years). In both groups, the retainers were bonded to all mandibular anterior teeth from canine to canine. All patients were recalled one year after bracket debonding. Randomization sequence was created using Excel 2010, with a 1:1 allocation, using random block size 4. The allocation sequence was concealed in sequentially numbered, opaque and sealed envelopes. Only participants were blinded to the type of bonded retainer used. The primary outcome was to compare the gingival condition between the two groups. The secondary outcomes were to assess plaque/calculus indices, irregularity index of the mandibular anterior teeth and retainers’ failure rate. Comparisons were conducted using Mann-Whitney U test or chi-square test. Statistical significance was predetermined at the *p*≤ 0.05 level for all tests.

**Results:**

Complete data were collected for 46 patients (round multi-strand wire retainer group, n=24 patients; rectangular Ortho-Flex-Tech™ retainer group, n=22 patients). No significant differences were found in the gingival health parameters between the two groups (*p*>0.05). Ortho-Flex-Tech™ retainers maintained the alignment of mandibular anterior teeth more than multi-strand retainer (*p*<0.05). No significant difference was found in the failure rate between the two groups (*p*>0.05).

**Conclusions:**

Gingival health parameters and failure rate were not different in both groups. However, Ortho-Flex-Tech™ retainers were more efficient to retain the mandibular incisors than the multi-strand retainers; nevertheless, the difference was not clinically significant.

## RESUMO

**Objetivo:**

O objetivo principal desse estudo foi comparar a saúde gengival após o uso de dois tipos de contenção: uma feita com fio redondo multifilamentado e outra feita com fio retangular Ortho-Flex-Tech™. Os objetivos secundários foram avaliar o acúmulo de placa/cálculo, determinar a eficácia dessas contenções em manter o alinhamento dos dentes, bem como sua taxa de falha.

**Material e Métodos:**

Esse foi um estudo unicêntrico do tipo ensaio clínico randomizado paralelo de dois braços, conduzido nas clínicas ortodônticas do Centro de Ensino Odontológico da *Jordan University of Science and Technology* (Jordânia). Foram selecionados aleatoriamente sessenta pacientes com contenção colada no segmento anterior inferior após tratamento ortodôntico fixo. A amostra foi composta por pacientes caucasianos com apinhamento pré-tratamento leve a moderado na região anterior inferior, relação de Classe I, tratados sem extração de dentes anteriores inferiores. Além disso, foram incluídos apenas os pacientes que apresentavam sobressaliência e sobremordida normais após o tratamento.

**Intervenção:**

Um grupo recebeu contenção com fio redondo multifilamentado (30 pacientes, idade média: 19,7 ± 3,8 anos), enquanto o outro grupo recebeu contenção com fio retangular Ortho-Flex-Tech™ (30 pacientes; idade média: 19,3 ± 3,2 anos). Em ambos os grupos, as contenções foram coladas em todos os dentes anteriores inferiores de canino a canino. Todos os pacientes retornaram um ano após a descolagem dos braquetes. A sequência de randomização foi criada no Excel 2010, com uma alocação de 1:1, usando tamanho de bloco aleatório 4. A sequência de alocação foi ocultada em envelopes numerados sequencialmente, opacos e lacrados. Apenas os participantes desconheciam o tipo de contenção usada. O resultado primário foi a comparação dos dois grupos quanto à condição gengival. Os resultados secundários foram a avaliação dos índices de placa/cálculo, índice de irregularidade dos dentes anteriores inferiores e taxa de falha das contenções. As comparações foram realizadas usando o teste U de Mann-Whitney ou o teste qui-quadrado. A significância estatística foi predeterminada ao nível de *p*≤ 0,05 para todos os testes.

**Resultados:**

Dados completos foram coletados para 46 pacientes (grupo de contenção com fio multifilamentado redondo, n=24 pacientes; grupo de contenção com fio retangular Ortho-Flex-Tech™, n=22 pacientes). Nenhuma diferença significativa foi encontrada entre os dois grupos para os parâmetros de saúde gengival (*p*>0,05). As contenções Ortho-Flex-Tech™ mantiveram mais o alinhamento dos dentes anteriores inferiores do que as contenções com fio multifilamentado (*p*<0,05). Nenhuma diferença significativa foi encontrada entre os dois grupos quanto à taxa de falha (*p*>0,05).

**Conclusões:**

Os parâmetros de saúde gengival e a taxa de falhas não foram diferentes entre os dois grupos. No entanto, as contenções Ortho-Flex-Tech™ foram mais eficientes em conter os incisivos inferiores do que as contenções com fio multifilamentado; no entanto, essa diferença não foi clinicamente significativa.

## INTRODUCTION

Retention of teeth after active orthodontic treatment is usually recommended to overcome the potential of relapse, which can be variable and unpredictable. A Cochrane review found a lack of high-quality evidence to favor one method of retention over another, in terms of stability.^1^ The duration of teeth retention has long been a dilemma in Orthodontics; however, long-term retention in the form of bonded retainer has been shown to be an effective way, in particular in the mandibular anterior segment, to minimize both relapse and maturational changes^2^ ^-^ ^4^ with minimal patient compliance.^5^ ^,^ ^6^

Two main designs of bonded retainers are currently in use: 1) rigid round wire bonded to the terminal teeth, which can be the first premolars or the canines; and 2) round multi-strand wire retainer bonded to all teeth in the anterior segment, usually from canine to canine.^7^ ^-^ ^9^ Multi-strand stainless steel wire retainers are increasing in popularity, due to their flexibility, which allows for some physiologic tooth movement.^10^

It has been reported that multi-strand wire retainers are more effective in maintaining individual tooth rotation, compared to the rigid wire retainers, whereas the latter were shown to be more hygienic.^11^ Furthermore, rigid wire retainers showed less failure rate, compared to the multi-strand wire retainers.^12^

Many systematic reviews^1^ ^,^ ^13^ ^-^ ^15^ found a lack of evidence to endorse the use of one type of orthodontic retainers based on their effect on: survival and failure rates, periodontal health, patient-reported outcomes and cost-effectiveness. Largely, these findings were attributed to a lack of high quality of relevant research and the high amount of methodological heterogeneity in study designs, types of wire used, methods of comparisons and outcomes reported.^14^ ^,^ ^15^

A new design of bonded retainer, called Ortho-Flex-Tech™, was recently developed by Reliance Orthodontic Products, Inc. (Itasca, IL, USA). The retainer is a low-profile chain-like design made of stainless steel or gold-plated (14 carat) stainless steel alloy, and usually bonded to the lingual surface of all mandibular anterior teeth, from canine to canine. This wire is rectangular in cross-section (0.974 x 0.402 mm / 0.0383 x 0.0158-in). The manufacturer claims that it has the advantages of easy application (naturally conforms to arch curvature), less chair time, low failure rate (flexible linkage), laboratory cost savings, and improved patient comfort (very low and flat profile). However, to the best of our knowledge, no previous studies have addressed the effectiveness of Ortho-Flex-Tech™ rectangular wire retainer in maintaining the alignment of teeth, its effect on the gingival health and the failure rate. 

Because the Ortho-Flex-Tech™ retainer is gold-plated and allow for physiologic tooth movement, it is thus hypothesized that it has a more hygienic design and promote less hazard on the gingival health. Additionally, the cross-section of the Ortho-Flex-Tech™ retainer is rectangular in shape, when compared to round multi-strand wire retainer, which could provide more tooth contact surface area and maintain the alignment of the teeth better than the round single point contact wire retainer. 

### SPECIFIC OBJECTIVES OR HYPOTHESES

The primary objective of this randomized clinical trial was to compare round multi-strand wires and Ortho-Flex-Tech™ rectangular wire retainers, in terms of gingival health. The secondary objectives were to assess plaque and calculus accumulation, and to determine the effectiveness of these retainers in maintaining tooth alignment, as well as their failure rate. The null hypothesis was that there would be no difference between the two retainers, regarding gingival health, plaque accumulation, tooth alignment and failure rate. 

## MATERIAL AND METHODS

### STUDY DESIGN AND CHANGES AFTER TRIAL COMMENCEMENT

This single-center study was a two-arm parallel randomized clinical trial with a 1:1 allocation. The methods were not changed after initiation of the trial.

### PARTICIPANTS, ELIGIBILITY CRITERIA AND SETTINGS

Ethical approval for the conduction of this study was obtained from the Institutional Research Board Committee (IRB) at King Abdullah University Hospital/ Jordan University of Science and Technology (JUST) in Irbid, Jordan. This clinical trial included 60 patients who were randomly selected from a pool of patients scheduled for debonding of orthodontic fixed appliances at the Dental Teaching Center/JUST, between 2015 and 2017, and who needed bonded retention for the mandibular anterior segment. Eligibility criteria included: (1) Caucasian patients with mild to moderate pretreatment crowding in the mandibular anterior region and Class I relationship, (2) the treatment plan did not involve extraction of mandibular anterior tooth, and (3) post treatment normal overjet and overbite. Exclusion criteria were: (1) missing mandibular anterior tooth, (2) history of previous orthodontic treatment, (3) spacing in the mandibular anterior region, (4) poor oral hygiene, (5) evidence of active periodontal disease. Participants of the study were selected based on the inclusion and exclusion criteria. After explaining the study implications, an informed consent was signed by the patient or the parent (in case of patients under 18 years of age).

All patients were treated using maxillary and mandibular pre-adjusted Edgewise fixed appliance (3M^®^Unitek, Victory Series, Monrovia, California, USA; 0.022-in slot; Roth prescription) by means of a non-extraction treatment protocol. 

### INTERVENTIONS

At the brackets debonding appointment, the appliances were removed, the teeth in all subjects were submitted to scaling by the same clinician (S.A.), and the retainers were bonded.

**Figure 1:**
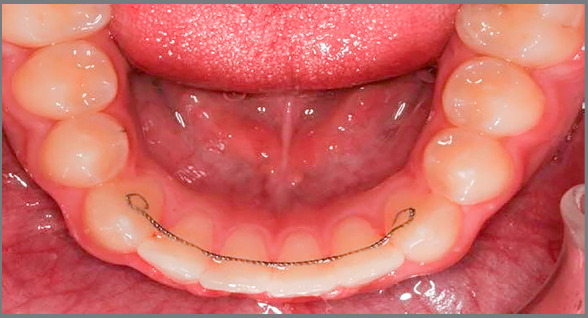
Multi-strand wire retainer bonded to the lingual surfaces of mandibular anterior teeth.

**Figure 2:**
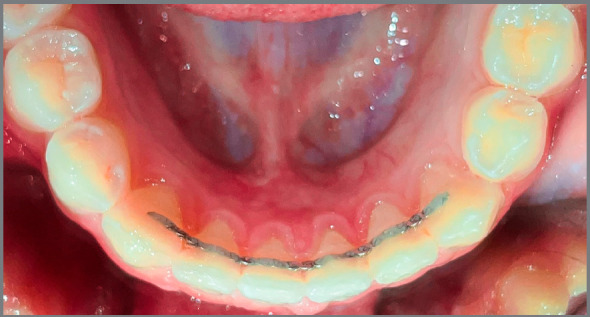
Ortho-Flex-Tech™ wire retainer bonded to the lingual surfaces of mandibular anterior teeth.

Both types of retainers were bonded to the lingual surfaces of all mandibular anterior teeth, from canine to canine, by the same clinician (S.A.), using Transbond LR composite (3M Unitek, Monrovia, California, USA). A standard procedure for fitting each bonded retainer was applied. For the multi-strand wire retainer group, a mandibular arch impression was taken and poured, to fabricate the final model for each patient. A multi-strand stainless steel wire (0.0215-in) retainer (3M Unitek, Monrovia, California, USA) was fitted to the model using rubber handles, which were then bonded to the lingual surface of the mandibular anterior teeth, to help holding the retainer while bonding (Fig 1). The Ortho-Flex-Tech™ rectangular wire (0.0383 x 0.0158-in) gold-plated stainless steel retainer was bonded directly to the lingual surface of the mandibular anterior teeth, according to the manufacturer instructions (Fig 2). Lingual surfaces were etched, rinsed and dried. Primer was then applied to all lingual surfaces, and a drop of Transbond LR composite was applied to every tooth’s lingual surface. Ortho-Flex-Tech™ retainer was then passively applied to all teeth from canine to canine. Oral hygiene instructions were given to all participants. Patients were asked to attend the clinic immediately within 24 hours, in case of any bonding failure or retainer fracture.

Simplified Oral Hygiene Index (SOHI) was assessed to compare the oral hygiene of the two groups. To measure the SOHI, four posterior and two anterior teeth surfaces were scored for debris and calculus accumulation, as described before by Greene and Vermillion.^16^ The six surfaces examined were the buccal surface of the maxillary first permanent molars, the lingual surface of the mandibular first permanent molars, the labial surface of the maxillary right and the mandibular left central incisors. In the present study, the mandibular left central incisor was excluded, as the amount of debris and calculus may have been affected by the presence of the bonded retainer. Debris amount was scored on a scale of 0 to 3. The total debris score was divided by the number of surfaces scored for each patient. The same method was used to obtain the Calculus Index scores. The debris score and calculus score were combined to obtain the SOHI score.

### OUTCOMES (PRIMARY AND SECONDARY) AND CHANGES AFTER TRIAL COMMENCEMENT

#### Primary outcome

One year after bracket debonding, all subjects were recalled by the same clinician (S.A.), and the Gingival Index (GI) was recorded for the mandibular anterior teeth. To obtain the GI, the buccal, lingual, mesial and distal surfaces of the mandibular anterior teeth were scored from 0 to 3; these scores were averaged to obtain the GI for each tooth.^17^ The total scores of the mandibular anterior teeth were divided by 4 to obtain the GI score for the mandibular anterior segment. 

#### Secondary outcomes

The secondary outcomes assessed the plaque index (PI), calculus index (CI), simplified oral hygiene index (SOHI), and irregularity index (IRI) of the mandibular anterior teeth, and retainers’ failure rate.

PI was determined by scoring the buccal, lingual, mesial and distal surfaces of the mandibular anterior teeth from 0 to 3, according to the amount of plaque on each surface.^17^ The total score was divided by 4 to obtain the PI for the tooth. The PI score for the mandibular anterior segment was the average PI score of the mandibular anterior teeth.

The IRI was determined by using Little’s irregularity index to measure the irregularity of the mandibular anterior teeth^18^. Contact point displacements were measured on digital casts generated from plaster models. Good quality alginate (Hydrogum from Zhermack Company, Badia Polesine - Italy) impressions were taken at the recall visit. Three-dimensional digital casts were generated from plaster models using Ceramill Map 400 scanner (Amann Girrbach, Koblach, Austria), which is accurate to 0.02mm. Contact point displacements were measured from the digital casts using Ceramill Mind design software (computer-aided design software, Amann Girrbach). The IRI for each subject was the sum of the measurements of the five contact points from canine to canine.

The patients were asked to attend the clinic immediately within 24 hours if the retainer was debonded from any tooth or if the retainer was broken. Moreover, the patients were recalled on monthly basis, to check for any broken retainer not perceived by the patient.

#### Sample size calculation

Sample size calculation was performed using G*power v. 3.1.9.4 software, based on a previous study^11^. The effect size was calculated as the mean difference between the two groups, divided by the standard deviation of one group. In a previous study,^11^the effect size for the gingival health and plaque indices was 0.46, whereas for the irregularity index, it was 0.96. The calculation revealed that 44 patients were required (22 patients per group) to achieve a power (1-β error) of 80% at alpha level of 0.05. Sixteen patients were added to compensate for attrition rate of 25%.

#### Interim analyses and stopping guidelines

Not applicable.

#### Randomization (random number generation, allocation concealment, implementation)

Participants were randomly allocated to either multi-strand wire or Ortho-Flex-Tech™ rectangular wire retainers. Randomization sequence was created using Excel 2010 (Microsoft, Redmond, WA, USA), with a 1:1 allocation, using random block size 4. Allocation concealment was applied before the trial commencement, to prevent selection bias. The allocation sequence was concealed in sequentially numbered, opaque and sealed envelopes from the investigator responsible for assigning participants into the intervention groups (K.A.), until the time of allocation implementation. Randomization sequence creation and allocation concealment were applied by another investigator (E.A.).

#### Blinding

Blinding of the investigator was not possible during clinical intervention or data measurement stage. Only participants were blinded to the type of bonded retainer used. The investigator (K.A.) responsible for the statistical analysis was blinded to the type of bonded retainer used in each group.

#### Statistical analysis (primary and secondary outcomes, subgroup analyses)

Statistical analysis was performed using the Statistical Package for the Social Sciences software (SPSS v. 22.0, SPSS Inc., IL, USA). Shapiro-Wilk w-test revealed that data were not normally distributed. Comparisons were conducted using Mann-Whitney U test or chi-square test; depending on the examined variable (numerical or categorical). Statistical significance was predetermined at the *p* ≤ 0.05 level for all tests.

#### Measurement error

To determine the measurement error, 10 subjects (5 subjects from each group) were randomly selected and re-examined by the same clinical examiner (S.A.) 7 days after the initial examination. The differences between first and second measurements were tested using the intraclass correlation coefficient for measurement error for the IRI.

## RESULTS

### PARTICIPANT FLOW

**Figure 3:**
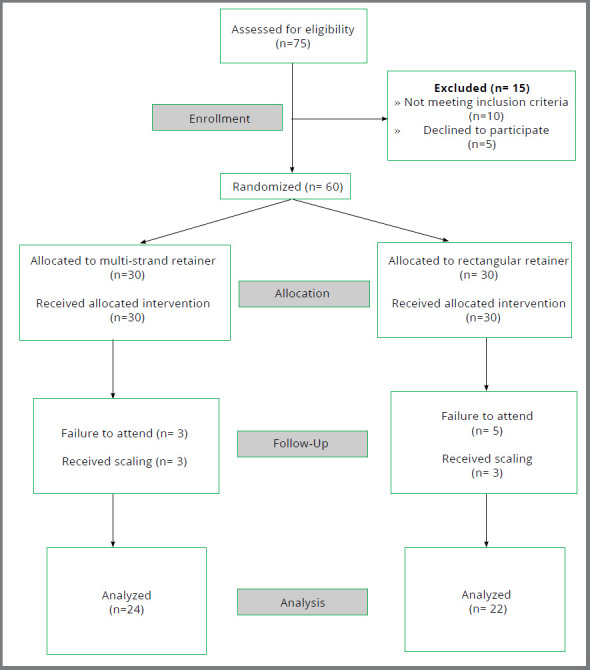
CONSORT flowchart showing the flow of participant data through the trial.

CONSORT flowchart showing the flow of participant data through the trial is presented in Figure 3. Sixty patients requiring bonded retention for the mandibular anterior segment were recruited from February 2015 to July 2017, with final data collection completed in July 2017. The sample was randomized in a 1:1 ratio to either retainer group (30 patients). Eight patients (13%) were excluded, as they failed to attend the clinic at the recall visit, and another six patients (10%) were excluded because they underwent scaling during the study period. Complete data were collected for 46 patients (Round multi-strand wire retainer group, n=24; Rectangular Ortho-Flex-Tech™ wire retainer group, n=22).

### BASELINE DATA

Baseline and pre-orthodontic treatment demographic and clinical characteristics for each group are presented in Table 1. 

**Table 1:** Baseline and pre-orthodontic treatment demographic and clinical characteristics for the two study groups.

	Multi-strand wire group n= 30	Ortho-Flex-Tech wire group n= 30	P-value	95% CI of the Difference
Age (years): Mean (SD)	19.2 (3.8)	19.7 (3.4)	0.74	-2.39-1.62
Gender (male/female)	8/22	10/20	0.76	-
Mandibular arch crowding (mm): Mean (SD) [95% CI]	4.7 (1.9) [4.05-5.35]	4.6 (1.5) [3.90-5.30]	0.5	-0.63-1.32
Mandibular anterior teeth irregularity (mm) Mean (SD) [95% CI]	7.6 (2.7) [6.75-8.45]	7.7 (2.7) [6.82-8.58]	0.78	-1.45-1.15
Maximum displacement in mandibular arch (mm) Mean (SD) [95% CI]	2.4(0.8) [2.15-2.65]	2.6(0.8) [2.32-2.88]	0.82	-0.51-1.28

*P*-value based on independent t-test. CI = Confidence Interval.

Baseline data for the gingival, calculus, plaque and simplified oral hygiene indices are expected to be deteriorated at the bracket debonding visit, due to fixed orthodontic treatment. Accordingly, the baseline data for these parameters were considered from the time of debonding; however, at this appointment, the teeth in all subjects were submitted to thorough scaling by the same clinician after bracket debonding, to improve these parameters and make the gingival health status for all included patients almost equal.

### NUMBERS ANALYZED FOR EACH OUTCOME, ESTIMATION AND PRECISION, SUBGROUP ANALYSES

Six (3 male, 3 females) of 30 patients from round Multi-strand wire retainer group, and 8 (6 male, 2 females) of 30 patients from Ortho-Flex-Tech™ wire retainer group were excluded from the study (Fig 3). The primary analysis was carried out per protocol, and complete data were obtained for 46 patients of the total 60 randomized sample: Multi-strand wire retainer group n=24, and Ortho-Flex-Tech™ wire group n=22.

### PRIMARY OUTCOMES

The mean GI scores for each group are shown in Table 2. There was no significant difference in GI between the two groups. The mean GI for different teeth surfaces is shown in Table 3. When the mean GI was divided into tooth surfaces, no significant differences were detected between the two groups.

**Table 2:** Comparison between Multi-strand wire (MS) and Ortho-Flex-Tech™ wire (OFT) retainer groups in terms of the gingival health parameters, IRI, and number of fractured retainers, standard deviation, 95% Confidence Interval, Median and Interquartile.

	MS group Mean (SD) [95% CI]	OFT group Mean (SD) [95% CI]	P-value	Median	25 - 75 quartiles	95% CI of the Difference
PI	0.51 (0.60) [0.26-0.76]	0.32 (0.51) [0.10-0.55]	0.157	0.104	0.00 - 0.60	-0.15 - 0.51
GI	0.93 (0.85) [0.58-1.29]	1.10 (0.63) [0.82-1.38]	0.459	1.15	0.33 - 1.69	-0.62 - 0.28
CI	0.16 (0.44) [-0.03-0.34]	0.25 (0.47) [0.04-0.46]	0.165	0.00	0.00 - 0.00	-0.36 - 0.18
SOHI	0.53 (0.52) [0.31-0.75]	0.42 (0.40) [0.24-0.59]	0.759	0.20	0.00 - 1.00	-0.16 - 0.39
IRI (mm)	0.69 (0.71) [0.51-0.87]	0.00 (0.00) -	0.048*	0.00	0.00 - 0.00	0.012 - 0.59
FR (n)	2	0	0.384	-	-	-

Mann-Whitney U test. Chi-square test (only for FR). * Significant (p < 0.05). PI: Plaque index. GI: Gingival index. CI: Calculus index. SOHI: Simplified oral hygiene index. IRI: Irregularity index. FR: Fractured Retainer.

**Table 3:** Comparison between Multi-strand wire (MS) and Ortho-Flex-Tech™ wire (OFT) retainer groups, in terms of the plaque index (PI), Gingival index (GI) and Calculus index (CI) of the mandibular anterior teeth. Means, standard deviation, 95% Confidence intervals, Median and interquartile.

	MS wire Mean (SD) [95% CI]	OFT wire Mean (SD) [95% CI]	P-value	Median	25 - 75 quartiles	95% CI of the Difference
PI buccal surface	0.17 (0.38) [0.01-0.33]	0.12 (0.31) [-0.01-0.26]	0.573	0.12	0.00 - 0.43	-0.36 - 0.29
PI lingual surface	0.61 (0.84) [0.26-0.96]	0.33 (0.71) [0.02-0.65]	0.091	0.13	0.00 - 0.47	-0.18 - 0.68
PI mesial surface	0.64 (0.74) [0.33-0.95]	0.43 (0.64) [0.15-0.72]	0.367	0.17	0.00 -0.88	-0.20 - 0.62
PI distal surface	0.61 (0.66) [0.33-0.89]	0.41 (0.52) [0.18-0.64]	0.223	0.25	0.00 - 0.71	-0.16 - 0.56
GI buccal surface	0.69 (0.78) [0.34-1.07]	0.49 (0.59) [0.18-0.76]	0.338	0.25	0.00 -1.13	-0.21 - 0.62
GI lingual surface	1.09 (0.78) [0.82-1.56]	1.46 (0.33) [1.24-1.68]	0.283	1.43	0.92 -1.67	-0.73 - 0.004
GI mesial surface	0.87 (0.79) [0.54-1.29]	1.11 (0.69) [0.84-1.48]	0.326	1.21	0.12 - 1.67	-0.68 - 0.21
GI distal surface	0.92 (0.96) [0.52-1.33]	1.14 (0.96) [0.71-1.56]	0.484	1.33	0.00 - 2.00	-0.78 - 0.36
CI buccal surface	0.00 (0.00)	0.00 (0.00)	1.000	0.00	0.00 - 0.00	-
CI lingual surface	0.21 (0.59) [-0.04-0.46]	0.27 (0.77) [-0.07-0.61]	0.880	0.00	0.00 - 0.00	-0.47 - 0.34

Mann-Whitney U test.

### SECONDARY OUTCOMES

Table 2 shows the SOHI, PI, and CI mean scores for each group. There was no significant difference in these parameters between the two study groups. The mean PI and CI for different teeth surfaces are shown in Table 3. When the mean PI and CI were divided into tooth surfaces, no significant differences were detected between the two groups. IRI scores and the number of fractured retainers for each group are shown in Table 2. The IRI was significantly higher in the Multi-strand wire group than the Ortho-Flex-Tech™ wire group one year after installing the retainer (*p*=0.048). However, the maximum mandibular incisor irregularity did not exceed 2 mm. The failure rate of multi-strand wire retainers was 8%, compared to zero failure rate of Ortho-Flex-Tech™ wire retainer group. However, this difference was not statistically significant (*p* > 0.05).

### ERROR OF THE METHOD

Intra-examiner reliability was very good, as the intraclass correlation coefficient for measurement error for the IRI was 0.884.

### HARMS

No negative outcomes were reported by any subject during the trial.

## DISCUSSION

Long-term retention of mandibular incisor alignment using bonded retainers is considered safe, predictable,^19^ acceptable to most patients and quite compatible with periodontal health.^5^ However, they undoubtedly may present some disadvantages and complications associated with the prolonged use, such as the adverse effect on periodontal health and failure of bonded retainer.

To the best of our knowledge, this randomized clinical trial is the first to compare Ortho-Flex-Tech™ wire and multi-strand wire retainer in terms of gingival health, plaque accumulation, tooth alignment and failure rate. 

Long-term retention with bonded retainers may present potential negative effects on periodontal health.^4^ In this study, PI, GI, CI, and SOHI were measured one year after debonding the appliances, since it has been reported that a minimum follow-up period of 6 months is necessary to distinguish between gingival inflammation associated with fixed orthodontic treatment and that related to the orthodontic retainers.^20^

No significant differences were detected between the two groups in this study in terms of PI, GI, CI, and SOHI. Even when the mean PI, GI and CI were divided into tooth surfaces, no significant differences were detected between the two groups. 

The gingival health and the plaque/calculus accumulation may have been affected by the subject’s oral hygiene status. Accordingly, it is important to have almost matched groups in terms of oral hygiene. In this study, baseline data for gingival parameters were considered at the time of bracket debonding, and not before orthodontic treatment or even during the active fixed orthodontic treatment. This can be considered as a limitation, since individuals with poor oral hygiene before or during treatment tend to have poor oral hygiene during retention phase. To reduce this bias, only patients with good oral hygiene were included in this study from the beginning. Also, randomization was performed to reduce the chance of unbalanced grouping. 

In this study, the PI (0.51) and GI (0.93), for multi-strand wire group were higher than that reported by Årtun et al.^21^ (PI = 0.13, GI = 0.39) and lower than that reported by Al-Nimri et al.^11^ (PI = 1.21, GI = 1.34). This difference may be related to patient attitude and general oral hygiene. No previous studies assessed impact of the Ortho-Flex-Tech wire retainer type on the gingival condition of the mandibular anterior segment.

A recent systematic review with meta-analysis^15^ concluded that orthodontic bonded retainers seem to be a retention strategy compatible with periodontal health, or at least not related to severe detrimental effects on the periodontium.

In the present report, the only statistically significant difference between the two groups was related to IRI. Ortho-Flex-Tech™ wire showed better alignment retention than multi-strand wire group. However, multi-strand wire group presented clinically less than 2 mm irregularity mean score. Previous studies have tried to define acceptable levels of relapse, and suggested that irregularity less than 3.5 mm in the anterior segment may be considered an acceptable level of relapse;^22^ ^,^ ^23^ however, some patients would not tolerate this minor amount of irregularity.^4^ Ortho-Flex-Tech™ wire retainer showed zero irregularity score, thus proving good efficiency. Ortho-Flex-Tech™ retainer has rectangular cross-sectional surface, compared to the round cross-sectional surface of the multi-strand wire retainer. This feature may give more control and prevent tooth from moving. However, a long-term study is still necessary to verify these results.

In the present study, the IRI of the multi-strand wire retainer group (0.69 mm) was almost similar to that reported by Årtun et al.^21^ (0.66 mm) and lower than that reported by Al-Nimri et al.^11^ (1.92 mm). No previous studies evaluated Ortho-Flex-Tech™ wire retainer in terms of maintaining the alignment. 

Although the difference in the retainers’ efficiency in this study was statistically significant, the clinical significance is questionable, as the mean difference in the IRI between the two groups was less than 1 mm. Long-term follow up may be necessary in the future to clarify this issue. 

Failure of a bonded retainer is a relatively common complication, with prevalence ranging from 1 to 53%.^24^ ^,^ ^25^ In this study, two retainers failed in the multi-strand wire group, compared to no failure in the Ortho-Flex-Tech™ group. However, this difference was not statistically significantly. Again, this may be attributed to the fact that both retainer types are bonded to all anterior teeth and present comparable flexibility, although they have different cross-sections and different bonding surface areas. 

The failure rate for multi-strand retainer (8%) was lower than that reported by Dahl and Zachrisson^26^ (20.6%), Årtun et al.^21^ (27.3%) and Al-Nimri et al.^11^ (29%). Interestingly, it was found that failure of fixed stainless steel mandibular retainers was not directly related to the duration of follow-up.^14^ This evidence suggests that other factors including the influence of operator technique and experience might override the effects of retainer design or materials.^14^

### LIMITATIONS

The following limitations are present in this study:

» The presence of high female to male ratio.
» This was a single-center study, performed by a single operator.
» The follow-up duration was relatively short (one year).
» Baseline data for gingival health parameters were considered at the time of bracket debonding.

## CONCLUSIONS

» No significant differences in the gingival health, plaque accumulation and calculus index were found between Ortho-Flex-tech™ wire and multi-strand wire groups.
» Ortho-Flex-tech™ wire retainer was significantly more efficient to maintain the alignment of mandibular anterior teeth than multi-strand wire retainer, although both retainers clinically showed satisfactory alignment maintenance.
» Although multi-strand wire retainers presented higher failure rate than Ortho-Flex-tech™ wire, this difference was not significant.
